# Selectively Targeting Leukemic MOLT-4 Cells by MTX-cIBR Conjugate: Mechanism of Action and Cellular Entry

**DOI:** 10.3390/life16060981

**Published:** 2026-06-11

**Authors:** Sista Werdyani, Meagan E. Weldele, Enade P. Istyastono, Sofia M. Harjana, Adi Hermawansyah, Wariya Nirachonkul, Dewi K. Paramita, Teruna J. Siahaan

**Affiliations:** 1 Study Program of Doctor in Biotechnology, Graduate School, Universitas Gadjah Mada, Jl. Teknika Utara, Kocoran, Caturtunggal, Depok, Sleman, Yogyakarta 55281, Indonesia; sista.werdyani@mail.ugm.ac.id; 2 Biomedical Laboratory, Department of Pharmacy, Faculty of Mathematics and Natural Sciences, Universitas Islam Indonesia, Jalan Kaliurang Km 14.5, Sleman, Yogyakarta 55584, Indonesia; 3 Simons Laboratories, Department of Pharmaceutical Chemistry, The University of Kansas, 2095 Constant Ave., Lawrence, KS 66047, USA; mweldele@jccc.edu; 4 Department of Pharmacy, School of Medicine and Health Sciences, Atma Jaya Catholic University of Indonesia, Jl. Jenderal Sudirman, Karet Semanggi, Jakarta 12930, Indonesia; enade.istyastono@atmajaya.ac.id; 5 Department of Histology and Cell Biology, Faculty of Medicine, Public Health and Nursing, Universitas Gadjah Mada, Jl. Farmako, Sekip Utara, Depok, Sleman, Yogyakarta 55281, Indonesia; sofia.mubarika@gmail.com; 6 Cell Culture Laboratory, Directorate of Innovation and Downstreaming, Universitas Muhammadiyah Yogyakarta, Jl. Brawijaya, Tamantirto, Kasihan, Bantul, Yogyakarta 55183, Indonesia; hermawansyah.adi@umy.ac.id; 7 Department of Medicinal Chemistry, The University of Kansas, 1251 Wescoe Dr, Lawrence, KS 66047, USA; wariya_n@ku.edu; 8 Research Center for Biotechnology, Universitas Gadjah Mada, Jl. Teknika Utara, Kocoran, Caturtunggal, Depok, Sleman, Yogyakarta 55281, Indonesia

**Keywords:** targeted delivery, leukemic T cell, MTX, cIBR, MTX-cIBR, MOLT-4, cell cycle, LFA-1, drug conjugates

## Abstract

The ICAM-1-derived cIBR peptide selectively binds to the I-domain of LFA-1, a receptor highly expressed on leukemia T cells; thus, the MTX-cIBR conjugate can be used to target methotrexate (MTX) to leukemic T cells and reduce its off-target toxicity. However, the uptake, biological mechanism, and selectivity of MTX-cIBR compared with unconjugated MTX remain unclear. Therefore, this study is aimed at evaluating the uptake, cytotoxicity, selectivity, apoptosis, cell cycle effects, and *DHFR*-related activity of MTX-cIBR in leukemia T cells compared with unconjugated MTX. MTX-cIBR exhibited cytotoxic activity comparable to MTX in LFA-1-expressing MOLT-4 cells but showed lower toxicity toward LFA-1-negative K562 cells, indicating improved selectivity. MTX uptake occurred through RFC and mFBP transport systems, whereas MTX-cIBR no longer depended on these pathways, suggesting altered cellular uptake after conjugation with cIBR by utilizing the LFA-1 receptor. Both compounds predominantly induced apoptosis with minimal necrotic cell populations. MTX induced S-phase arrest at lower concentrations and G2/M induced arrest at higher concentrations, whereas MTX-cIBR consistently promoted S-phase accumulation. In addition, MTX and MTX-cIBR downregulated the expression of *DHFR*, *FPGS*, and *TYMS* in MOLT-4 cells. Computational analyses further demonstrated that MTX exhibited lower binding free energy (ΔG) and greater binding stability toward *DHFR* than MTX-cIBR. These findings suggest that MTX-cIBR retains selective cytotoxic activity toward LFA-1-expressing leukemia T cells through altered cellular uptake and exhibits different interaction characteristics with *DHFR* compared with unconjugated MTX.

## 1. Introduction

Cancer is the second most prevalent non-communicable disease globally. Leukemia is a hematological malignancy that accounts for 3.2% of cancer cases and 3.9% of cancer-related mortality in the US [1]. Various methods to treat cancer include surgery, radiation, and chemotherapy; however, many chemotherapeutic agents produce severe side effects such as nausea, vomiting, gastrointestinal discomfort, diarrhea, and constipation [2,3,4,5]. Therefore, there is a need to develop methods to lower the side effects of chemotherapeutics. One way to lower the side effects is by targeting the chemotherapeutic agent to cancer cells but avoiding its delivery to normal cells. Targeted drug delivery strategies have been successfully used to treat cancer patients by conjugating anticancer drugs to peptides and monoclonal antibodies (mAbs) that recognize a specific target receptor on the cancer cells [6,7,8]. For example, antibody–drug conjugates (ADCs) such as Adcetris (brentuximab vedotin) and Kadcyla^®^ (trastuzumab emtansine, T-DM1) have been successfully used to treat patients with Hodgkin’s lymphoma (HL) and breast cancer, respectively [6,9]. Similarly, peptide–drug conjugates (PDCs) have emerged as a promising method to direct chemotherapeutic and diagnostic agents (theranostics) to cancer cells while sparing healthy tissues in patients [10,11,12,13]. In this case, OctreoScan^®^ is an FDA-approved tumor diagnostic agent; it is an octreotide (fCFyKTCT) peptide conjugated to diethylenetriaminepentaacetic acid (DTPA) complexed with ^111^In to generate ^111^In-DTPA [13].

Leukemic T cells (i.e., Molt-4) express the α_L_β_2_ integrin receptor that is also called the lymphocyte function-associated antigen-1 (LFA-1) receptor. The LFA-1 receptor binds to the intercellular adhesion molecule 1 (ICAM-1) protein that is expressed in most cells, including immune cells. The ICAM-1/LFA-1 interactions serve as a mediator of T-cell adhesion to target cells (i.e., antigen-presenting cells, epithelial and endothelial cells), and this interaction also acts as a co-stimulatory signal for T-cell activation. Peptides derived from the domain-1 (D1) of ICAM-1, such as cIBR cyclic peptide, cyclo(1,12)PenPRGGSVLVTGC, bind to the I-domain of the LFA-1 receptor to block T-cell adhesion to ICAM-1-expressing endothelial and epithelial cells [14,15,16,17,18,19,20]. In addition, the cIBR peptide is internalized by LFA-1 into leukemia cells (i.e., HL-60 and MOLT-3) via receptor-mediated endocytosis process through endosomes and finally resides in the lysosomes. In contrast, endothelial cells that do not express LFA-1 cannot endocytose the cIBR peptide; thus, the cIBR peptide can be used to selectively target drugs to leukemic T cells [15,17,21,22].

**Figure 1:**
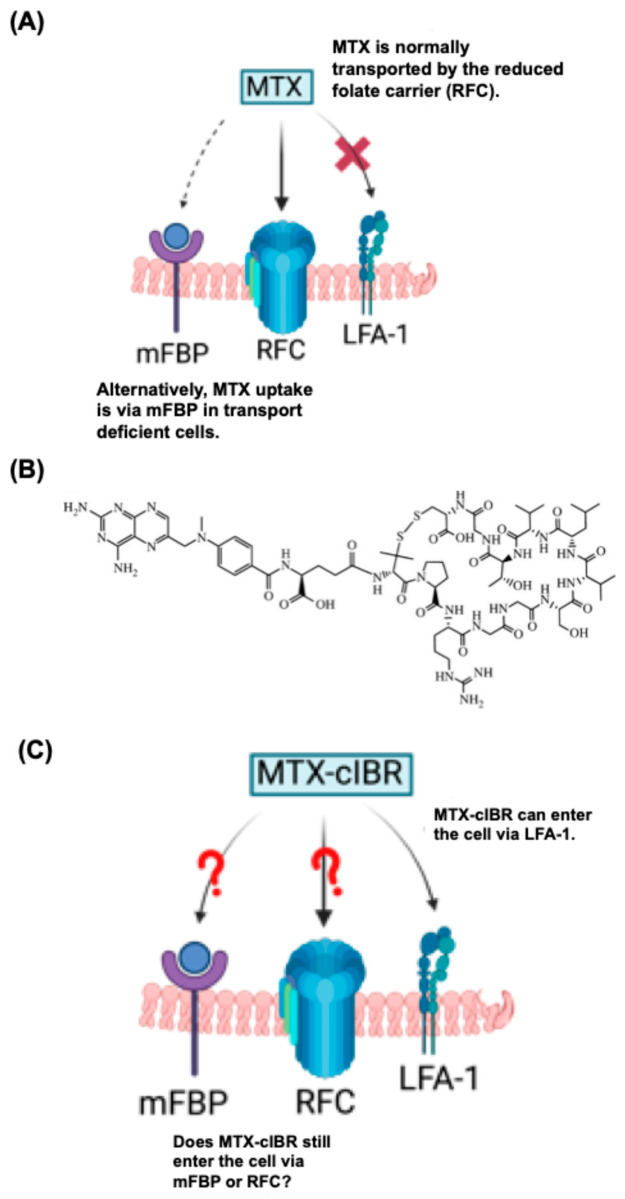
(**A**) The cellular uptake mechanisms of MTX via RFC and mFBP. (**B**) Chemical structure of MTX-cIBR. (**C**) The potential cellular uptake mechanisms of MTX-cIBR conjugate via LFA-1, RFC, and mFBP. Figure 1A,C were created in BioRender. Werdyani, S. (2026) https://BioRender.com/dcrh5tc.

Methotrexate (MTX) is a commonly used chemotherapeutic agent for leukemia at high doses. At low doses, MTX has also been used to treat rheumatoid arthritis (RA). MTX is an orally bioavailable drug and can enter the cells via the reduced folate carrier (RFC), which is a folic acid transporter expressed in both cancerous and normal cells (Figure 1A). It can also enter the cell via membrane folate-binding proteins (mFBP; Figure 1A). Inside the leukemia and normal cells, MTX inhibits the dihydrofolate reductase (*DHFR*) enzyme that is important in DNA synthesis. As a result, MTX stops cell proliferation by inducing cellular damage [23]. Due to the uptake mechanisms into both cancer and normal cells, MTX can have side effects in patients, which include fatigue, diarrhea, nausea, hair loss, as well as liver and kidney damage. Thus, there is a need to target MTX to leukemia T cells by forming an MTX-cIBR conjugate that targets the LFA-1 receptor but avoids normal cells (Figure 1B) [24,25]. The hypothesis is that MTX-cIBR is targeted to LFA-1-expressing MOLT-4 cells with reduced uptake by RFC and mFBP to avoid non-LFA-1-expressing cells (Figure 1C).

Previously, the MTX-cIBR conjugate was shown to kill LFA-1-expressing MOLT3 cells; this activity can be inhibited by the cIBR peptide to confirm that the LFA-1 receptor still recognizes the cIBR fragment in MTX-cIBR [26]. Additionally, MTX-cIBR has been shown to suppress RA in the rat adjuvant model with lower toxicity than MTX [10]. Although previous studies demonstrated the LFA-1-targeting capability of MTX-cIBR in MOLT-3 cells, its detailed biological mechanism and selectivity compared with unconjugated MTX remain unclear. In this current study, the selectivity of MTX-cIBR was compared to MTX in killing LFA-1-expressing MOLT-4 T cells and LFA-1-non-expressing K562 cells [16,20,27]. MOLT-4 cells were selected as an LFA-1-expressing leukemia T-cell model based on previous reports describing LFA-1 expression in T-cell leukemia models and cIBR-mediated targeting of MOLT cells, whereas K562 cells were used as an LFA-1-negative control cell line [28,29].

The involvement of RFC and mFBP in MTX-cIBR uptake was investigated using the L1210-WT murine leukemia cell line together with its variants, L1210-1565 and L1210-FBP (Figure 1C). L1210-1565 cells are deficient in RFC transport activity and were used to evaluate RFC-mediated uptake, whereas L1210-FBP cells overexpress membrane folate-binding protein (mFBP) and were used to assess mFBP-mediated transport. By comparing MTX and MTX-cIBR responses in these cell lines, we aimed to determine whether conjugation with cIBR alters the conventional uptake pathways of MTX. The mechanism of toxicity of MTX-cIBR was compared to MTX as a comparative reference and positive control; in this case, the apoptosis mechanism and the cell cycle arrest induced by these molecules were determined. The effects of MTX-cIBR and MTX in suppressing dihydrofolate reductase (*DHFR*), folylpolyglutamate synthase (*FPGS*), and thymidylate synthase (*TYMS*) expressions were investigated.

## 2. Materials and Methods

### 2.1. Materials

MTX used in this study was purchased from Merck, Darmstadt, Germany (catalog no. PHR1396), while MTX-cIBR was synthesized following the previous method by Majumdar et al. [10]. The purity and identity of MTX-cIBR were analyzed by analytical reversed-phase high-performance liquid chromatography (RP-HPLC) (Supplementary Figure S1A) and matrix-assisted laser desorption/ionization time-of-flight mass spectrometry (MALDI-TOF MS) (Supplementary Figure S1B). Previous studies reported that MTX-cIBR exhibits the greatest stability under near-physiological conditions (pH 6–7), supporting its stability during the cell culture experiments performed in this study [10]. All solvents used for analysis were of analytical grade and purchased from Fisher Chemical (Waltham, MA, USA). The MOLT-4 (catalog # CRL-1582) and K562 (catalog # CCL-243) cell lines were obtained from ATCC (Rockville, MD, USA) and maintained for a limited number of passages after thawing (no more than 10 passages) to minimize phenotypic variation. L1210-1565 and L1210-WT mouse leukemia cell lines were obtained from the Institute for Cancer Research (London, UK), and L1210-FBP cells were a kind gift from Dr. Gerrit Jansen (University Free Hospital, Amsterdam, Netherlands). These three cells were also maintained for a limited number of passages after thawing (no more than 10 passages). All the cell culture media for the in vitro assays were purchased from Gibco (Grand Island, NY, USA). The Roswell Park Memorial Institute (RPMI)-1640 medium was supplemented with 10% fetal bovine serum, 1% penicillin/streptomycin, and 0.5% amphotericin B. The in silico analysis was performed using YASARA Structure version 24.4.10, running on Windows 11 Home Single Language (version 14H2) with an 11th Gen Intel^®^ Core™ i5-1135G7 with a 2.40 GHz processor.

### 2.2. Cell Lines

MOLT-4 cells were originally derived from a patient with T-cell acute lymphoblastic leukemia, while K562 cells originated from a patient with chronic myelogenous leukemia and are classified as erythroleukemia cells. L1210-WT is a drug-sensitive murine cell line originally derived from spontaneous lymphocytic leukemia in a DBA/2 mouse. Meanwhile, L1210-1565 is a subline of L1210 cells with altered sensitivity to antifolate drugs and is commonly used to study antifolate resistance. L1210-mFBP is another L1210 variant that overexpresses membrane-associated folate-binding proteins (mFBP). All cell lines were cultured under standardized conditions and used during the logarithmic growth phase for all experiments. Upon receipt, the cells were thawed in the supplemented RPMI-1640 (Gibco, Grand Island, NY, USA) medium and cultured in a humidified incubator at 37 °C in a 5% CO_2_ environment. Once the cells reached confluency, they were harvested and prepared for subsequent assays using RPMI-1640 medium containing 10% FBS.

### 2.3. Cytotoxic and Selectivity Analysis Using MTT-Assay

The cytotoxicity and selectivity of MTX-cIBR and MTX were compared using an MTT assay. Both cell lines were seeded in 96-well plates at a density of 2 × 10^4^ cells/well and incubated overnight. The following day, five different concentrations of each compound were added to the wells. MOLT-4 cells were treated with 0.25, 0.5, 1.0, 2.0, and 4.0 μM, whereas K562 cells were treated with 12.5, 25, 50, 100, and 200 μM. The final DMSO concentration in all treatments did not exceed 0.2%. After 24 h of incubation, 3-(4,5-dimethylthiazol-2-yl)-2,5-diphenyltetrazolium bromide (MTT, Merck, Darmstadt, Germany) was added at a final concentration of 0.5 mg/mL. The plates were then incubated for an additional 4 h, followed by the addition of a stop solution containing 10% sodium dodecyl sulfate (SDS; Merck, Darmstadt, Germany). After overnight incubation, absorbance was measured at 570 nm using a SpectraMax Plus microplate reader (Molecular Devices, San Jose, CA, USA). All experiments were performed in triplicate and independently repeated at least three times. The IC_50_ values were calculated using GraphPad Prism software version 1.0.2 (100) (GraphPad Software Inc., San Diego, CA, USA) by nonlinear regression analysis using a normalized dose–response curve with variable slope, followed by calculation of the selectivity index (SI = IC_50_ K562/IC_50_ MOLT-4).

### 2.4. Apoptosis Assay

Apoptosis analysis in this study was performed by incubating MOLT-4 cells for 24 h in a CO_2_ incubator. Cells were seeded in 6-well plates at a density of 5 × 10^5^ cells/well. Cells were then treated with MTX-cIBR and MTX at three different concentrations for 24 h. After treatment, cells were harvested and separated from the medium by centrifugation. The resulting pellets were washed twice with phosphate-buffered saline (PBS) before adding 5 µL Annexin V (Thermo Fisher Scientific, Waltham, MA, USA) and 10 µL propidium iodide (PI, Thermo Fisher Scientific, Waltham, MA, USA), followed by incubation for 20 min. Cells were then washed twice with PBS and finally resuspended in 300 µL PBS. The prepared cell suspension was subsequently analyzed by the Cytek^®^ Aurora flow cytometer (Cytek Biosciences, Fremont, CA, USA). The gating strategy for apoptosis analysis was established using untreated control cells. Initial gating was performed using FSC versus SSC plots to exclude debris and identify the main cell population. Singlet cells were then selected using FSC-A versus FSC-H plots to exclude doublets and cell aggregates. The singlet population was subsequently analyzed based on Annexin V-FITC and PI fluorescence to distinguish viable, early apoptotic, late apoptotic, and necrotic cell populations.

### 2.5. Cell Cycle Analysis

Confluent MOLT-4 cells were seeded in 6-well plates at a density of 5 × 10^5^ cells/well and incubated for 24 h at 37 °C under 5% CO_2_. After incubation, MTX-cIBR and MTX at several different concentrations were added to each well, followed by incubation for 24 h, with the same conditions. Following treatment, the cells were centrifuged, and the resulting pellets were washed twice with PBS, followed by fixing the cells with 200 µL of 70% cold ethanol at 4 °C for 30 min. Then, the cells were washed again twice with PBS, followed by staining with 10 µL of PI for 20 min at room temperature. Finally, the cell pellets were resuspended in 300 µL of PBS for cell cycle analysis using the NovoCyte Advanteon flow cytometer (Agilent Technologies, Santa Clara, CA, USA). The gating strategy for cell cycle analysis followed the same initial procedure as the apoptosis assay, including FSC versus SSC gating to exclude debris and FSC-A versus FSC-H gating to select singlet cells. The resulting singlet population was then analyzed based on PI fluorescence intensity to determine the distribution of cells in the G0/G1, S, G2/M, and sub-G1 phases.

### 2.6. RFC and mFBP Transport of MTX and MTX-cIBR

L1210 and L1210-1565 cells were seeded at a density of 5 × 10^4^ cells/well in 900 µL of culture medium in 24-well plates. After 4 h of incubation, 110 µL of serially diluted MTX or MTX-cIBR was added to the treatment wells, while an equivalent volume of medium was added to the control wells. Cells were then incubated for 48 h (L1210 cells) or 72 h (L1210-1565 cells), corresponding to approximately four cell divisions. Cell growth inhibition was determined by cell counting using a Z2 Coulter Counter (Coulter Electronics Ltd., Luton, Beds, UK). The IC_50_ values for each compound were calculated using GraphPad Prism software version 1.0.2 (100) (GraphPad Software Inc., San Diego, CA, USA). The cross-resistance (CR) value [30] for each compound was calculated by dividing the IC_50_ value obtained in L1210-1565 cells by that obtained in L1210 wild-type (WT) cells. As L1210-1565 cells lack functional RFC, whereas L1210-WT cells possess normal RFC activity, the CR value reflects the extent of RFC-mediated transport and has been widely used as an indicator for this purpose [31].

To evaluate the potential involvement of the mFBP in mediating the transport of MTX-cIBR compared to MTX, a cell growth inhibition assay was performed using the L1210-FBP cell line. In these experiments, the effect of an mFBP blockade was assessed by the addition of excess folic acid (FA). FA was prepared in an unsupplemented medium, sterile-filtered, and added to L1210-FBP cell suspensions 15 min prior to compound treatment to achieve a final concentration of 1 µM FA. L1210-FBP cells without FA supplementation were plated in parallel as controls. Serial dilutions of MTX or MTX-cIBR were added to both FA-treated (FA^+^) and untreated (FA^−^) cells, followed by incubation under standard culture conditions for 72 h, allowing approximately four cell divisions. Growth inhibition was determined as described above, and IC_50_ values under FA^+^ and FA^−^ conditions were calculated using GraphPad Prism software version 1.0.2 (100) (GraphPad Software Inc., San Diego, CA, USA).

### 2.7. Gene Expression Using qRT-PCR

Confluent MOLT-4 cells were seeded in 6-well plates at a density of 2.5 × 10^5^ cells per well and incubated for 24 h prior to treatment with MTX or MTX-cIBR at three different concentrations: 1, 10, and 100 μM. Following a 48 h incubation (longer incubation was needed to allow a detectable change in mRNA expression), the total RNA was extracted from the treated cells using the Invitrogen™ PureLink™ (Waltham, MA, USA) RNA Mini Kit, following the manufacturer’s protocol. On the same day, the isolated RNA was immediately reverse transcribed into cDNA using the Thermo Scientific™ (Waltham, MA, USA) RevertAid First Strand cDNA Synthesis Kit, according to the manufacturer’s instructions. The concentration and purity of both RNA and cDNA were determined using a UV spectrophotometer (Cary 100 Bio UV-Vis, Agilent, Mulgrave, Australia) by measuring absorbance at 260 and 280 nm.

To evaluate gene expression, real-time PCR amplification was performed. The target genes selected were those directly involved in the mechanism of action of MTX involving *DHFR*, *FPGS*, and *TYMS*. The housekeeping gene Glyceraldehyde-3-Phosphate Dehydrogenase (*GAPDH*) was used as an internal control. Primers for all four genes were obtained from the previously published literature (Table 1). To ensure primer specificity, primer alignment analysis was conducted using NCBI tools. Once verified, the primers were synthesized by Eurofins. Primer efficiency was evaluated using standard curve analysis, and amplification specificity was confirmed by melt curve analysis, where a single melting peak indicated the specific amplification of the target product.

Quantitative real-time PCR was carried out using the QuantStudio™ (Waltham, MA, USA) 5 Real-Time PCR System. The PCR protocol included 40 cycles consisting of denaturation at 95 °C, annealing at 60 °C, and extension at 72 °C. The reaction mixture was prepared using Applied Biosystems™ Power SYBR™ Green PCR Master Mix (Thermo Scientific, Waltham, MA, USA), in accordance with the manufacturer’s protocol. Gene expression was analyzed using the system’s built-in software and quantified using the ΔΔCt parameters. The expression levels were normalized to *GAPDH* as the internal reference.

### 2.8. Molecular Docking and Molecular Dynamics (MD) Simulations

The *DHFR* structure was obtained from the Protein Data Bank (PDB ID: 4M6K) via https://www.rcsb.org/structure/4M6K (Accessed on 20 May 2025). Folic acid was used as a reference ligand to validate the docking system by performing 100 docking runs with *DHFR*. The system was considered valid when the RMSD values of all docking results were less than 2 Å. Molecular dynamics simulations were then performed to evaluate the stability of the poses using the method described by Liu et al. [36]. Once stable poses were confirmed, docking experiments were carried out for MTX and MTX-cIBR following the same procedure. Finally, the binding free energy (ΔG) was calculated for both molecules and compared with the published K_m_ and V_max_ values of MTX and MTX-cIBR with *DHFR* [26].

### 2.9. Statistical Analysis

Statistical analyses were performed using GraphPad Prism software version 1.0.2 (100) (GraphPad Software Inc., San Diego, CA, USA). IC_50_ values were calculated by nonlinear regression analysis using a normalized dose–response curve with variable slope. Comparisons between two groups were analyzed using an unpaired Student’s t-test, whereas multiple group comparisons were evaluated using one-way analysis of variance (ANOVA) followed by Tukey’s post hoc test. Data are presented as mean ± standard deviation (SD) from at least three independent experiments. The differences were considered statistically significant at *p* < 0.05. In all figures, * indicates *p* < 0.05; ** indicates *p* < 0.01; *** indicates *p* < 0.001; and **** indicates *p* < 0.0001.

## 3. Results

### 3.1. Compound Analysis of MTX-cIBR

The MTX-cIBR conjugate (Figure 1B) was synthesized by conjugating the γ-carboxyl group of MTX with the N-terminus of cIBR peptide as previously described [10]. RP-HPLC analysis in the C18 column for MTX-cIBR showed a single peak at 13 min, indicating its purity (Supplementary Figure S1A). The MALDI-TOF MS spectrum showed a desired molecular weight of 1610 Da for MTX-cIBR with two small additional peaks [M + Na^+^] and [M + K^+^] (Supplementary Figure S1B). These analytical results were similar to those found previously by Majumdar et al. [10].

### 3.2. Cytotoxicity and Selectivity of MTX-cIBR Compared to MTX in MOLT-4 Cells

**Figure 2:**
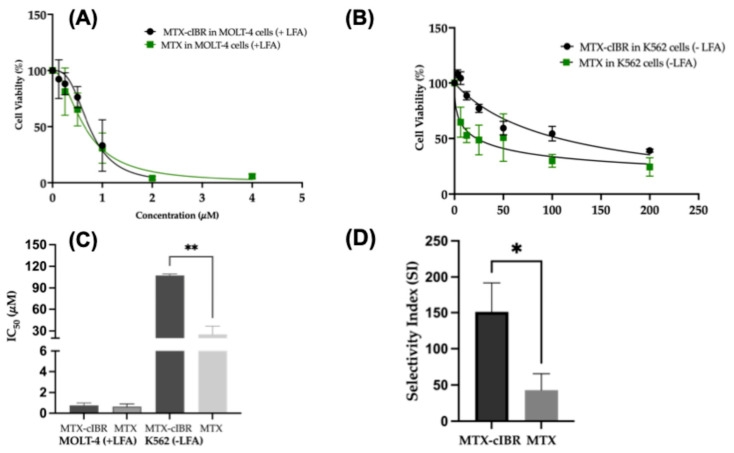
Cytotoxicity and selectivity of MTX and MTX-cIBR in MOLT-4 (LFA positive (+)) and K562 (LFA negative (−)) cells. (**A**) Dose–response curves in MOLT-4 cells showed that both MTX and MTX-cIBR reduce cell viability in a dose-dependent manner. (**B**) A similar trend was observed in K562 cells, but only at significantly higher concentrations. (**C**) IC_50_ values were generally lower in MOLT-4 cells for both MTX-cIBR and MTX. MTX exhibits higher cytotoxicity than MTX-cIBR in K562 cells. (**D**) Selectivity index analysis indicated that MTX-cIBR is more selective toward LFA-1-positive cells compared to unconjugated MTX. * indicates *p* < 0.05. ** indicates *p* < 0.01.

MTX-cIBR was designed to target LFA-1-expressing cells such as leukemic MOLT-4 T cells, but it should not target K562 cells that do not express LFA-1 receptors. Thus, the concentration-dependent toxicities of MTX-cIBR and MTX were compared in MOLT-4 cells and K562 cells using 3-(4,5-dimethylthiazol-2-yl)-2,5-diphenyltetrazolium bromide (MTT) assay (Figure 2). The data showed a dose-dependent response with lower viable cells at higher doses of drugs. MTX-cIBR and MTX have similar toxicity against MOLT-4 T cells with IC_50_s of 0.75 ± 0.23 and 0.64 ± 0.25 µM, respectively (Figure 2A). In contrast, MTX (IC_50_ = 25.18 ± 11.48 µM) has higher toxicity than MTX-cIBR (IC_50_ = 107.33 ± 2.05 µM) in K562 cells (Figure 2B). The IC_50_ data indicate that both MTX-cIBR and MTX were more toxic in MOLT-4 T cells than in K562 cells; however, MTX was more toxic than MTX-cIBR in K562 cells (Figure 2C). The selectivity index (SI) for both molecules was determined using the ratio between the IC_50_ in K562 over the IC_50_ in MOLT-4 (SI = IC_50_ K562/IC_50_ MOLT-4). MTX-cIBR has a higher specificity toward MOLT-4 than K562, with an SI = 151 compared to MTX with an SI of 43 (Figure 2D). This is presumably due to the selective uptake of MTX-cIBR via the LFA-1 receptor on MOLT-4 cells compared to non-LFA-1 expressing K562 cells. These results were consistent with previous studies that showed MTX-cIBR can be internalized by LFA-1-expressing cells, and the internalization can be blocked by cIBR peptide and anti-α-subunit of LFA-1 [10,37].

### 3.3. Apoptosis Analysis of MOLT-4 Cells After Treatment with MTX-cIBR and MTX

**Figure 3:**
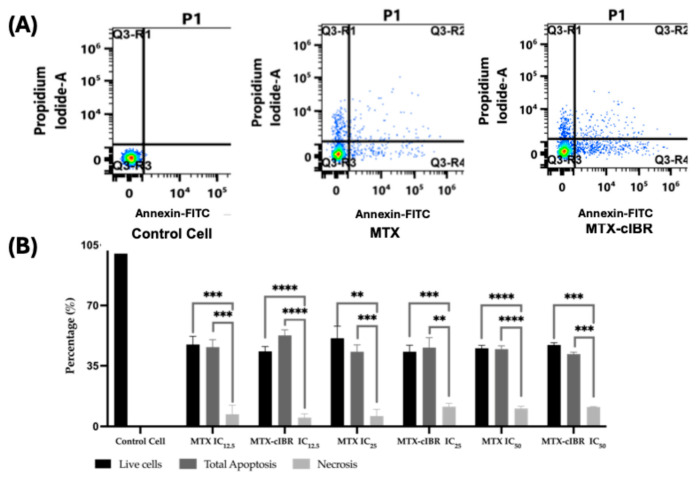
Apoptosis analysis by flow cytometry using FITC-labeled Annexin V and PI staining. (**A**) Dot plots of Annexin V-FITC (X-axis) vs. PI (Y-axis) showed similar patterns for both MTX and MTX-cIBR, indicating predominant apoptosis cell death. (**B**) The proportion of apoptotic cells was higher than that of necrotic cells in both treatments, with no significant difference between MTX and MTX-cIBR at the same doses. ** indicates *p* < 0.01; *** indicates *p* < 0.001; and **** indicates *p* < 0.0001.

The effects of MTX-cIBR and MTX were compared in inducing apoptosis and necrosis mechanisms in MOLT-4 cells at three different concentrations (i.e., IC_12.5_, IC_25_, and IC_50_). These mechanisms were detected by flow cytometry after cell staining with PI (Y-axis) and Annexin V (X-axis) (Figure 3A and Supplementary Figure S3). This dual-staining approach allows discrimination between viable cells, early apoptotic cells, late apoptotic cells, and necrotic cells based on membrane integrity and phosphatidylserine externalization. FITC-Annexin V binds to phosphatidylserine (PS) that is flipped to the outer leaflet of the membranes. The lower left quadrant (PI^−^/Annexin V^−^) represents viable cells. The upper left quadrant (PI^+^/Annexin V^−^) represents necrotic cells. The lower right quadrant (PI^−^/Annexin V^+)^ represents early apoptotic cells. The upper right quadrant (PI^+^/Annexin V^+^) represents late apoptotic cells. In the control group, almost all cells were viable. Both MTX- and MTX-cIBR-treated cells were distributed across all four quadrants. The quantitative distributions of cells for each population are shown in Figure 3B.

Because the primary objective was to determine the proportion of cells undergoing necrosis versus apoptosis, cells in both early and late apoptotic stages were combined and defined as total apoptosis. Both MTX and MTX-cIBR predominantly induced apoptotic cell death across all three different doses. No substantial shift toward necrotic cell death was observed when the dose was increased from IC_12.5_ to IC_50_. Thus, the cytotoxicity of both MTX and MTX-cIBR was mainly driven by their pharmacological action rather than by external or environmental stress. At all three doses of MTX and MTX-cIBR, the necrotic populations are significantly lower than the apoptosis populations, as well as the viable cell populations. Similarly, the viable cell populations of MTX and MTX-cIBR at all conditions were similar to the apoptosis populations.

### 3.4. The Effects of MTX and MTX-cIBR on the Cell Cycle of MOLT-4 Cells

**Figure 4:**
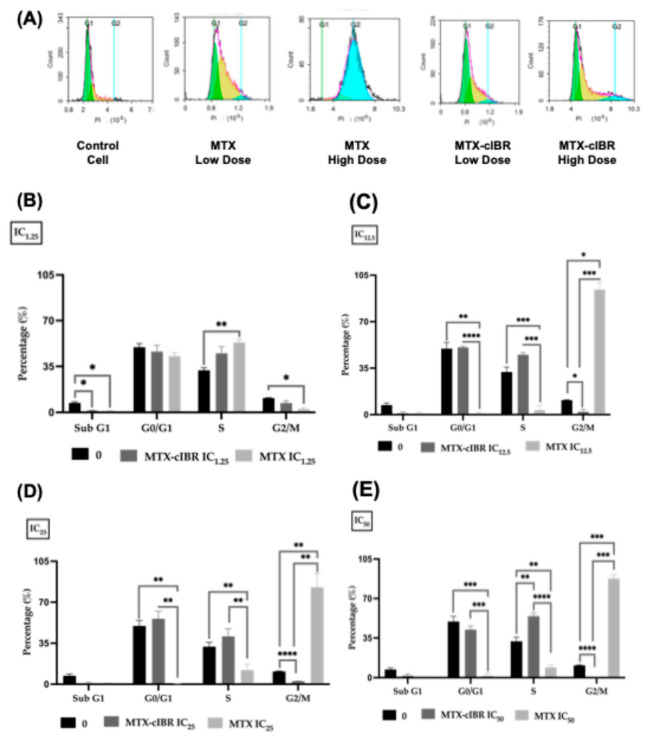
The effects of different doses of MTX and MTX-cIBR on cell cycle regulation of MOLT-4 cells by flow cytometry using PI staining. (**A**) Cell cycle regulation profiles following treatment with different doses of MTX and MTX-cIBR showed distinct cell cycle distribution patterns, such as Sub G1, G0/G1, S-phase, and G2/M cell cycle arrest positions. (**B**–**E**) Cell cycle arrest populations of MOLT-4 induced by MTX and MTX-cIBR at different doses: (**B**) IC_1.25_, (**C**) IC_12.5_, (**D**) IC_25_, and (**E**) IC_50_. * indicates *p* < 0.05; ** indicates *p* < 0.01; *** indicates *p* < 0.001; and **** indicates *p* < 0.0001.

Cytotoxic drugs, including MTX, that induce apoptosis are often closely linked to cell cycle regulation, as several apoptotic regulatory proteins are functionally interconnected with key cell cycle regulators [38]. Therefore, this study was aimed at comparing the impact of MTX-cIBR and MTX on cell cycle progression. An increased percentage of cells at a given phase compared to the untreated control group indicates the inhibition at that stage; thus, the cells were unable to proceed to the next phase of the cell cycle. The flow cytometry profiles of the control and treatment groups were distinct, and the two treatment groups also exhibited different patterns (Figure 4A).

At the IC_1.25_ concentration, MTX and MTX-cIBR treatment produced a significantly lower Sub-G1 population compared to the control group (Figure 4B). Similarly, there are trends of lower Sub-G1 populations on cells treated with MTX and MTX-cIBR at higher concentrations (i.e., IC_12.5_, IC_25_, IC_50_) compared to the control (Figure 4C–E). At the IC_1.25_ concentration, there was no difference in the G0/G1 phase population between control cells and cells treated with MTX and MTX-cIBR (Figure 4B). At concentrations higher than IC_1.25_, there were significantly lower populations of G0/G1 phase for cells treated with MTX compared to untreated control and MTX-cIBR-treated cells (Figure 4C–E). There were no differences in the G0/G1 population between MTX-cIBR-treated cells and control cells at all concentrations (Figure 4B–E). This suggests that cells were still able to progress to the next phase of the cell cycle, namely the S phase. At the lowest dose of IC_1.25_, MTX-treated cells have a high population in the S-phase (Figure 4B), but the S-phase population drops significantly lower than the control at IC_12.5_, IC_25_, and IC_50_ concentrations (Figure 4C–E). In contrast, MTX-cIBR has a high population at the S-phase at all doses, suggesting that MTX-cIBR predominantly inhibits cell cycle progression at the S phase (Figure 4B–E). In contrast, MTX shifted cells to the G2/M phase arrest at higher concentrations (i.e., IC_12.5_, IC_25_, and IC_50_) (Figure 4C–E).

### 3.5. RFC and mFBP Transport of MTX and MTX-cIBR

To evaluate the involvement of RFC in transporting MTX-cIBR, the cross-resistance (CR) value of this conjugate was compared to that of MTX in L1210-WT and L1210-1565 [30]. The L1210-WT is a wild-type cell that has a high expression of RFC and low expression of mFBP. In contrast, the L1210-1565 is an MTX-resistant cell that lacks functional RFC and has low mFBP expression. Using these two types of cells, the CR value can be calculated by dividing the IC_50_ values of the drug in L1210-1565 over L1210-WT cells (Table 2) [30]. The data showed that MTX has a lower IC_50_ in L1210-WT than in L1210-1565 cells, with the CR value of 93, which is consistent with a previously reported CR value of 96 [31]. This indicates that MTX is transported into L1210-WT cells by RFC. In contrast, MTX-cIBR has the same IC_50_ in both L1210-WT and L1210-1565 cells with a CR value of 1.01. In addition, MTX-cIBR has 392-fold higher IC_50_ than MTX in L1210-WT, while MTX-cIBR has only 4.2-fold higher IC_50_ than MTX in L1210-1565 cells [39]. Overall, the data indicated that MTX-cIBR is not transported by an RFC receptor.

For MTX, the alternative transport mechanism is via the mFBP receptor that can be evaluated using the L1210-FBP cell line; this cell has mFBP overexpression with reduced expression of RFC. In this case, the transport of MTX and MTX-cIBR by mFBP can be blocked by the presence of excess folic acid (FA) due to their competition for mFBP. The transport sensitivity of MTX and MTX-cIBR in the presence of FA was assessed by calculating the ratio of the IC_50_ value in the presence of FA (FA+) and in the absence of FA (FA−) (Table 3) [40]. In the presence of FA, the IC_50_ values of MTX increased 90-fold (sensitivity value), indicating that the reduced cellular uptake was due to the competition between MTX and FA for the mFBP receptor. This sensitivity index in the presence of FA is consistent with a previously reported value of 73 [41]. In contrast, the IC_50_ values of MTX-cIBR increased 1025-fold in the presence of FA, suggesting that MTX-cIBR binding to the mFBP receptor is weaker than MTX. In summary, MTX-cIBR was not effectively transported by mFBP compared to MTX in the L1210-FBP cell line [40].

### 3.6. Gene Expression Analysis in MOLT-4 Cells upon Treatment with MTX and MTX-cIBR

**Figure 5:**
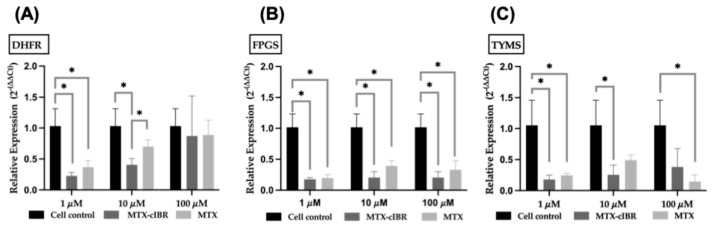
Gene expression analysis in MOLT-4 cells using qRT-PCR after treatment with MTX and MTX-cIBR at different concentrations: 1, 10, and 100 μM. The observed gene expression changes are found in (**A**) *DHFR*, (**B**) *FPGS*, and (**C**) *TYMS*. * indicates *p* < 0.05.

The effects of MTX and MTX-cIBR on gene expressions of primary and secondary target enzymes for MTX activity were determined, including dihydrofolate reductase (*DHFR*), folylpolyglutamate synthase (*FPGS*), and thymidylate synthase (*TYMS*). Compared to control, treatment of MOLT-4 cells with MTX significantly suppressed *DHFR* expression at 1.0 µM but not at 10 and 100 µM concentrations (Figure 5A). It is interesting to find that there is a trend of increased *DHFR* expression as the concentrations of MTX increase. Similarly, MTX-cIBR treatment significantly suppressed *DHFR* expression at 1.0 and 10 µM but not at 100 µM (Figure 5A). Similar to MTX, a trend of increasing DHFR expression was observed as the concentration of MTX-cIBR increased. At 10 µM, the *DHFR* expression was significantly higher in MTX-treated cells than in the MTX-cIBR-treated cells.

Treatments of cells with MTX and MTX-cIBR at three different doses significantly suppressed the expression of *FPGS* (Figure 5B). *FPGS* has a role for polyglutamation of folic acid (FA) and MTX to produce FA-(Glu)_n_ and MTX-(Glu)_n_ molecules that are trapped in the intracellular space of the cell. There were no significant differences in the FGPS expression when treated with both MTX and MTX-cIBR at three different concentrations, indicating that both molecules regulate *FPGS* expression through a similar mechanism. There was a trend of suppression of *TYMS* expression in MOLT-4 when treated with MTX and MTX-cIBR compared to the control (Figure 5C). However, all three concentrations of MTX-cIBR did not show any statistically significant reduction relative to the control group. Only MTX-treated cells at 100 µM showed statistically significant suppression of *TYMS* expression.

### 3.7. Predicted Interaction of DHFR with MTX and MTX-cIBR in YASARA Software

The optimal binding properties of MTX and MTX-cIBR to *DHFR* enzyme were evaluated using YASARA docking with default parameters for local search. In YASARA docking, the reported binding free energy (ΔG) represents the predicted stability of the ligand–protein complex (Table 4). This value is primarily derived from the VINA local search (VINALS), which estimates the overall binding affinity based on a scoring function. Adaptive Poisson–Boltzmann Solver (APBS) provides complementary information by calculating the electrostatic potential and solvation effects, helping to clarify which energetic contributions drive the binding (Table 4). While these values cannot directly yield kinetic parameters such as K_m_ or V_max_, they serve as proxies for binding affinity and inhibition strength, offering mechanistic insight that complements the experimental assays. MTX has a lower binding free energy (ΔG) than MTX-cIBR, suggesting that MTX has stronger binding than MTX-cIBR to *DHFR* (Table 4). These ΔG values are consistent with the K_m_ values of *DHFR* enzyme activity after treatment with MTX and MTX-cIBR [26]. The reported K_m_ for MTX was 1.84 ± 0.7 × 10^−9^ M, while the K_m_ for MTX-cIBR was more than 20-fold higher (29.8 ± 2.1 × 10^−9^ M). Overall, MTX-cIBR is a weaker inhibitor of *DHFR* than MTX.

**Figure 6:**
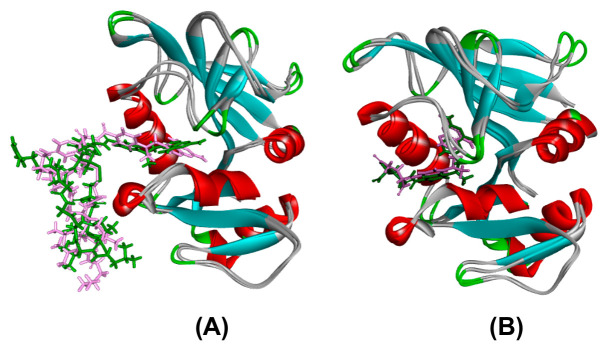
Visualization of molecular docking results (green) superimposed with molecular dynamics results (pink) for (**A**) MTX-cIBR and (**B**) MTX in *DHFR.* The results show that MTX exhibits a better fit within the enzyme active site compared to MTX-cIBR.

For MTX, the molecular docking and molecular dynamics (MD) results after 100 docking runs showed one dominant pose (97%). This pose reached equilibrium after 5 ns during the MD equilibration, and in the first MD production run, a stable pose was identified. The simulation was repeated three times, after which the ΔG was calculated. For MTX-cIBR, two representative clusters were found with the lowest predicted energy. Cluster 01, positioned at the MTX binding pocket, while Cluster 02, with the second-best binding energy, had the highest number of poses (35 poses). MTX-cIBR exhibited a more distributed interaction pattern at the active site of *DHFR* (Figure 6A), and it forms hydrogen bonds with residues Arg33, Gln36, Gly46, Gln48, Lys69, Val116, Glu45, and Thr40, as well as hydrophobic interactions with Phe35, Phe32, Arg37, Arg33, Ile61, and Leu68, as well as π–cation interactions with Lys69 and Arg71 (Figure 6A). This widespread interaction profile suggests that MTX-cIBR does not fit deeply into the canonical binding pocket but instead interacts with multiple peripheral residues, likely due to steric hindrance from the bulky cIBR moiety. Consistently, MD simulations revealed reduced stability of MTX-cIBR, with a tendency to deviate from the binding pocket over time. In contrast, MTX is accommodated within the binding pocket, forming multiple stabilizing interactions with surrounding residues and the cofactor (Figure 6B). MTX was found to form hydrogen bond interactions with Ser60, Pro67, and Lys69, along with a hydrophobic interaction with Phe32 and a π–cation interaction with NADPH (NAP202), indicating its proper orientation within the binding pocket and favorable interaction with the cofactor. These interactions are consistent with a stable binding mode where MTX binds to the active side with stable interactions during MD simulations.

## 4. Discussion

Targeting a toxic drug, such as MTX, to a specific cancer cell can improve its efficacy by lowering its side effects in a patient [10]. Conversely, if a drug can indiscriminately enter both cancerous and normal cells, it can induce cytotoxic effects in normal cells, causing adverse side effects that compromise patient comfort and reduce treatment compliance. In this study, MTX-cIBR selectively targets LFA-1-expressing MOLT-4 cells over non-LFA-1-expressing K562 cells (Figure 1C), and it has the potential to reduce the adverse effects of MTX [34]. As a control, MTX and MTX-cIBR have very low toxicity in human dermal fibroblast cells, with cell viability remaining close to 100% at all tested concentrations (Supplementary Figure S2). Previously, in Molt-3 cells, the uptake and cytotoxicity of MTX-cIBR can be blocked by anti-LFA-1 (CD11a) mAb or cIBR peptide in a dose-dependent manner [14]. Therefore, conjugation of cIBR to FITC and MTX to make FITC-cIBR and MTX-cIBR, respectively, did not interfere with the binding properties of the conjugates to LFA-1 receptors on the surface of T cells [17,26]. Colocalization study using confocal microscopy revealed that FITC-cIBR binds to the α-subunit of LFA-1 on the surface of T cells [17]. NMR studies indicated cIBR peptide binds to the I-domain α-subunit of LFA-1 by inserting the G5-S6-V7-L8-V9-T10-G11 segment into the I-domain allosteric site (IDAS) binding pocket [43]. In this case, the N- and C-termini of the cIBR peptide are exposed to the solvent [43]. Thus, it is reasonable to expect that conjugating FITC or MTX to the N-terminus of cIBR does not interfere with the binding of FITC-cIBR or MTX-cIBR to the IDAS binding pocket of LFA-1 on the surface of leukemic T cells (i.e., MOLT-4, MOLT-3).

Because LFA-1 can internalize FITC-cIBR, it is expected that MTX-cIBR can also undergo receptor-mediated endocytosis by LFA-1 receptors on T cells. The activity of MTX-cIBR on MOLT-3 cells can be inhibited by cIBR peptide and anti-I-domain mAb, indicating that MTX-cIBR was internalized by LFA-1 in MOLT-3 T cells [26]. The endocytosis process of FITC-cIBR goes through early and late endosomes and finally resides in the lysosomes for its degradation in MOLT-3 and HL-60 cells [17,21]. Therefore, MTX-cIBR undergoes a similar endocytosis process as FITC-cIBR, followed by degradation of MTX-cIBR in the lysosomes, presumably via a catabolism mechanism to release MTX that inhibits *DHFR* activity inside the cell. This proposal is supported by the similar IC_50_s of both MTX-cIBR and MTX in MOLT-4 T cells (Figure 2A). If the intact MTX-cIBR causes the *DHFR* inhibition, the IC_50_ of MTX-cIBR should be higher than the IC_50_ of MTX. This is because the affinity of MTX-cIBR is lower (K_m_ = 29.8 ± 2.1 × 10^−9^ M) than MTX (K_m_ = 1.84 ± 0.7 × 10^−9^ M) in an isolated *DHFR* enzyme [26], suggesting that the intact MTX-cIBR should have lower toxicity than MTX in MOLT-4 T cells.

Molecular docking and molecular dynamics simulations indicated that MTX was tightly bound within the active-site pocket of *DHFR*, maintaining a stable interaction for at least 5 ns. MTX formed hydrogen bond interactions with Ser60, Pro67, and Lys69, together with a hydrophobic interaction with Phe32 and a π–cation interaction with NADPH (NAP202), supporting its favorable accommodation within the DHFR binding pocket. In contrast, MTX-cIBR did not bind as tightly to the active pocket, and it appeared to drift from the MTX binding site, suggesting that it has lower binding stability compared with MTX (Table 4). The lower *DHFR* affinity of MTX-cIBR than MTX is due to the presence of the cIBR moiety in MTX-cIBR that can interfere with the binding of its MTX fragment at the *DHFR* active site, as shown in the in silico analysis (Figure 6) [10]. Unlike MTX, MTX-cIBR exhibited a broader interaction profile involving hydrogen bond interactions with Arg33, Gln36, Gly46, Gln48, Lys69, Val116, Glu45, and Thr40, as well as hydrophobic interactions with Phe35, Phe32, Arg37, Arg33, Ile61, and Leu68. In addition, π–cation interactions with Lys69 and Arg71 were observed. This distributed interaction pattern suggests that MTX-cIBR may interact more extensively with peripheral residues surrounding the binding region rather than being deeply accommodated within the canonical active pocket.

MTX-cIBR has two binding poses, where the first pose has the MTX segment inserted into the *DHFR* active site. The second binding pose has lower energy than the first pose, but it does not incorporate the MTX segment into the active site. This finding may indicate an altered binding mode for MTX-cIBR compared with MTX and suggests the possibility of non-canonical or peripheral interactions with *DHFR*. In these cases, binding the free energy (ΔG) of MTX-cIBR to *DHFR* was higher compared to that of MTX (Table 4); these results support the lower affinity of MTX-cIBR compared to MTX as reflected by their K_m_s. This observation supports the suggestion that MTX-cIBR activity inside the cells is due to the cleavage of MTX in the lysosomes to inhibit the *DHFR* because MTX-cIBR has a similar IC_50_ to MTX in MOLT-4 cells.

Normally, cancer cells can evade cell death by activating survival mechanisms that prevent them from entering programmed cell death pathways, allowing continuous proliferation and cellular immortality. During early apoptosis, phosphatidylserine—which is normally located on the inner leaflet of the plasma membrane—is externalized and can be detected by Annexin V. In contrast, during necrosis, loss of membrane integrity causes cellular DNA to leak out of the cell and be detected by propidium iodide (PI). Accordingly, Annexin V-positive cells are considered apoptotic, whereas PI-positive cells indicate necrotic cell death (Figure 3A). Cells that show a negative response for both Annexin V and PI are classified as viable cells. Cells with a positive response to both Annexin V and PI represent late apoptotic cells, in which membrane integrity is lost, allowing PI to enter and bind to DNA [44]. Across all concentrations and treatments for both MTX and MTX-cIBR, total apoptosis was significantly higher than necrosis (*p* < 0.001), but the total apoptosis was not significantly different from the viable cell population (*p* > 0.05) (Figure 3). Both MTX and MTX-cIBR predominantly induced apoptosis in MOLT-4 cells (Figure 3); however, MTX can trigger necrosis at a high dose. Comparisons between MTX and MTX-cIBR at equivalent concentrations showed no statistically significant differences in any cell population across all doses (*p* > 0.05). These results also support the suggestion that the activity of MTX-cIBR is due to the release of MTX from MTX-cIBR degradation in the lysosome.

The apoptosis pathway is a regulated cell death (RCD) process that is modulated by pharmacological or genetic interventions [45,46,47]; drug-induced cancer cell death is normally via apoptosis as the dominant pathway rather than necrosis. Thus, MTX or MTX-cIBR-induced apoptosis was driven by intrinsic factors within the cell rather than by external environmental factors; this confirms that the MTX-cIBR action is due to its internalization into the cell by LFA-1 [48]. The necrosis pathway is an accidental cell death (ACD) that occurs rapidly in response to severe physical, chemical, or mechanical stresses (e.g., extreme temperature, pressure, pH variation, or shear force) [45,46,47]. Moreover, necrotic cell death is typically associated with inflammatory responses within cells, while apoptosis represents a regulated, non-inflammatory form of programmed cell death [49,50]. It is well established that apoptosis can be triggered by cell cycle arrest involving several regulatory proteins in the signaling pathways. Furthermore, prolonged or irreversible cell cycle arrest can ultimately lead to apoptosis [38,51]. MTX-cIBR and MTX block the cell cycle in the S-phase; however, this arrest shifts to the G2/M phase when treated with MTX at a high dose (Figure 4). G2/M phenotype usually arises under drug combinations or post-release from an S-phase block, as reported in studies describing G2/M accumulation after MTX washout and in MTX–pretubulysin regimens [52,53]. In another finding, inducing cell cycle arrest in G2/M can occur because the cells are resistant to the drug, which is similar to the MOLT-4 cell response to higher doses of MTX (Figure 4C–E) [54]. Unlike MTX at higher doses, MTX-cIBR did not induce the G2/M cycle at higher doses (Figure 4C–E), indicating that MTX-cIBR may not induce resistance in MOLT-4 cells.

The gene expressions of primary and secondary target enzymes for MTX were evaluated in the effects of MTX and MTX-cIBR treatments. The inhibition of these enzymes suppresses purine and pyrimidine biosynthesis, which is needed for DNA synthesis to cause cell death [54,55]. For normalization, Glyceraldehyde-3-Phosphate Dehydrogenase (*GAPDH*) was used as the housekeeping gene to calculate the relative gene expression levels. The function of *DHFR* is to reduce dihydrofolate to tetrahydrofolate for purine and thymidylate synthesis. At low concentrations (i.e., 1–10 µM), MTX-cIBR and MTX suppressed mRNA expression of *DHFR* compared to control (Figure 5A); however, the mRNA expression of *DHFR* was increased, similar to the control, at 100 µM of MTX and MTX-cIBR. It is interesting to observe that there was a trend of increase in mRNA expression of *DHFR* as the dose of MTX and MTX-cIBR was increased. This result suggests that the increase in mRNA expression is the result of the resistant response of MOLT-4 cells to the increase in concentration of MTX inside the cell. There was also a trend that the mRNA expression was lower in cells treated with MTX-cIBR compared to MTX at 1–10 µM, suggesting that MTX-cIBR generated less resistance than MTX alone. Overall, the increase in mRNA expression of *DHFR* is presumably due to a resistant response to a higher concentration of MTX inside the cell, and this resistance could also be the reason why the MTX also induces cells into a G2/M cell cycle arrest at a higher dose.

*FPGS* is the key enzyme responsible for converting the MTX into its active polyglutamated form, MTX-(Glu)_n_. As a type 1 prodrug, MTX is metabolized by *FPGS* into a polyglutamate derivative such as MTX-(Glu)_n_, which are the active forms with a long residence time inside the cell [55,56]. Patients treated with MTX show a high concentration of MTX-(Glu)_n_ up to 80%, as well as up to 25% in the DBA/1J mice as a collagen-induced arthritis model [57]. This indicates that MTX-(Glu)_n_ is a good marker for MTX treatment, with its dose-dependent accumulation in erythrocytes [57]. MTX-(Glu)_n_ is the potent form that inhibits *DHFR*, *TYMS*, 5-aminoimidazole-4-carboxamide ribonucleotide transformylase (AICART), and amido-phosphoribosyl transferase enzymes. At low to high concentrations (1.0–100 μM), both MTX and MTX-cIBR suppressed the mRNA expression of *FPGS* and *TYMS* compared to control (Figure 5B,C). The expression levels of *FPGS* and *TYMS* did not differ significantly between MTX and MTX-cIBR across the three tested doses. Unlike *DHFR*, the increase in concentrations of MTX and MTX-cIBR did not increase the mRNA expressions of *FPGS* and *TYMS*.

**Figure 7:**
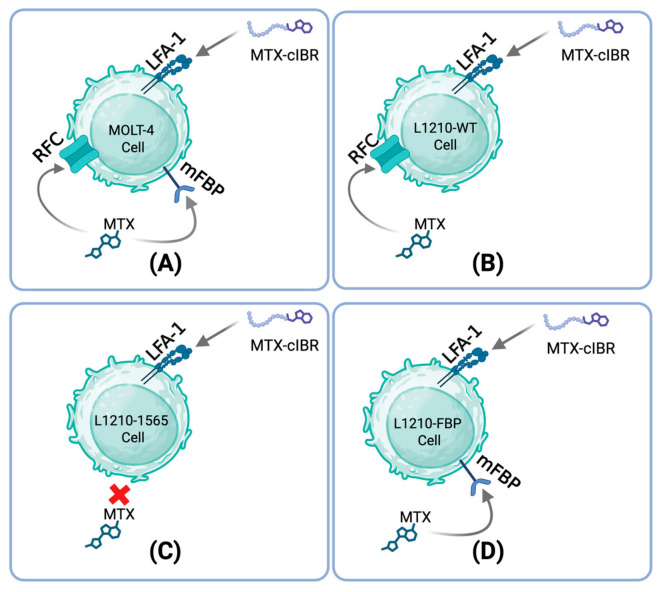
Proposed cellular uptake mechanisms of MTX and MTX-cIBR in (**A**) MOLT-4, (**B**) L1210-WT, (**C**) L1210-1565, and (**D**) L1210-FBP cells based on the experimental findings. The results suggest that MTX uptake primarily occurs through RFC- and mFBP-mediated transport pathways, whereas MTX-cIBR uptake is mainly mediated by LFA-1. Figure 7 was created in BioRender. Werdyani, S. (2026) https://BioRender.com/ep0nml8.

Although MTX-cIBR was selective toward LFA-1-expressing leukemic T cells such as Molt-3, MOLT-4, and HL-60 cells, MTX-cIBR can also be internalized by transporters for MTX such as RFC and mFBP (Figure 7). This current study has shown that MTX-cIBR was not efficiently internalized by RFC because it has the same IC_50_s in L1210-WT cells with high expression of RFC and in L1210-1565 cells with low expression of RFC; the CR value of MTX-cIBR is 1.1 (Table 2). In contrast, MTX has a lower IC_50_ in L1210-WT than in L1210-1565 cells with a CR value of 93 (Table 2). Similarly, MTX-cIBR has a lower affinity to mFBP compared to MTX because MTX-cIBR can be easily inhibited by FA compared to MTX in L1210-FBP cells with a high expression of mFBP. Mechanistically, this finding is consistent with the structural and functional properties of RFC as a transporter for folate via a channel-like mechanism driven by anion exchange [58,59]. Given the relatively large size of MTX-cIBR, it is most likely that it cannot traverse through the RFC transport channel.

Although MTX-cIBR and related cIBR-based conjugates have previously been evaluated in vivo in rheumatoid arthritis mouse models, the present study was limited to in vitro leukemia cell models. Therefore, additional in vivo studies using leukemia models are required to further evaluate the therapeutic efficacy, selectivity, pharmacokinetics, and safety profile of MTX-cIBR under physiological conditions. Furthermore, time-dependent cytotoxicity experiments should be carried out in the future to provide more detailed information regarding the kinetics of MTX-cIBR uptake, intracellular processing, and cytotoxic response compared with MTX. cIBR derivatives with fewer amino acids to minimize steric hindrance have been developed to further improve the efficiency of synthesis as well as formation of their conjugates [14,22]. The modification was focused on reducing the amino acid sequences of linear peptides such as LH_4_ (VILPRG) and LH_7_ (PRGGSV), as well as their respective cyclic forms, CH_4_ (Cyclo(1,6)VILPRG and CH_7_ (Cyclo(1,6)PRGGSV). These derivatives can inhibit the ICAM-1/LFA-1-mediated T-cell adhesion [14]. The cyclic peptide cIBR7 (cyclo(1,8)CPRGGSVC) has been successfully conjugated to FITC and doxorubicin (DOX) to make FITC-cIBR7 and Dox-cIBR7; these conjugates were internalized by LFA-1 on the surface of HL60 leukemic cells [22]. In the future, MTX-CH_7_ and MTX-cIBR7 will be evaluated for their selectivity and mechanisms of action in killing leukemic T cells over normal cells [18,22].

## 5. Conclusions

In summary, MTX-cIBR has the potential to be developed as a targeted drug delivery strategy for leukemia T cells, providing selective cytotoxicity with reduced off-target effects, while differing from MTX in its mechanisms of cell cycle regulation, *DHFR* expression, and predicted binding affinity. In the future, the binding affinity of cIBR peptide derivatives will be improved by optimizing their amino acid sequence to include D amino acids and unnatural amino acids.

## Figures and Tables

**Table 1:** The list of primers used to analyze the expression of selected target genes related to methotrexate activity.

Gene	Primer Forward (5′-3′)	Primer Reverse (5′-3′)	Reference
***GAPDH***	TTGGCTACAGCAACAGGGTG	GGGGAGATTCAGTGTGGTGG	[32]
***DHFR***	CACAACCTCTTCATAGAAGGTAA	CTGCCACCAACTATCCAGAC	[33]
***FPGS***	ATGGAGTACCAGGATGCCGT	GGCTTCCAACTGTGTCTGAG	[33]
***TYMS***	CGGGAGACATGGGCCTCGGT	GCATCCAGCCCAACCCCTAA	[34,35]

**Table 2:** RFC-mediated transport in L1210 cells.

Compound	IC_50_ Value (μM)		Cross Resistance [30] L1210-1565/L1210-WT
	L1210-WT	L1210-1565	
MTX	0.014 ± 0.0004	1.3 ± 0.64	93
MTX-cIBR	5.5 ± 2.0	5.6 ± 1.0	1.01

**Table 3:** mFBP-mediated transport in L1210-FBP cells.

Compound	IC_50_ Value (μM)		Sensitivity (FA+/FA−)
	With 1 μM FA	Without FA	
MTX	0.54 ± 0.06	0.006 ± 0.0078	90
MTX-cIBR	4.1 ± 2.3	0.004 ± 0.001	1025

**Table 4:** Predicted binding free energy (ΔG) values from docking analysis.

Rep	MTX		MTX-cIBR	
	APBS (kcal/mol)	VINALS (kcal/mol)	APBS (kcal/mol)	VINALS (kcal/mol)
1	72.077	−9.198	80.415	−7.330
2	63.180	−10.137	63.436	−7.134
3	67.495	−9.208	61.647	−7.662
Mean	67.584	−9.514	68.499	−7.375
SD	4.449	0.539	10.358	0.267

Note: the +/− signs obtained from YASARA were reversed to follow chemical convention, as YASARA originally applies the physical convention [42].

## Data Availability

The data presented in this study are available on request from the corresponding author.

## Supplementary Materials

The following supporting information can be downloaded at https://www.mdpi.com/article/10.3390/life16060981/s1. Figure S1. HPLC traces and mass spectrometry data for MTX-cIBR. Figure S2. Cell viability (%) of human dermal fibroblast cells following treatment with MTX and MTX-cIBR. Figure S3. Representative flow cytometry plots of apoptosis analysis in MOLT-4 cells treated with MTX and MTX-cIBR.

## Abbreviations

The following abbreviations are used in this manuscript:

AICART	5-aminoimidazole-4-carboxamide Ribonucleotide Transformylase
APBS	Adaptive Poisson–Boltzmann Solver
ATCC	American Type Culture Collection
CO_2_	Carbon Dioxide
CR	Cross-Resistance
Da	Dalton
*DHFR*	Dihydrofolate Reductase
DNA	Deoxyribonucleic Acid
FBS	Fetal Bovine Serum
FITC	Fluorescein Isothiocyanate
*FPGS*	Folylpolyglutamate Synthase
G2/M	Gap 2/Mitosis
*GAPDH*	Glyceraldehyde-3-phosphate Dehydrogenase
IC_12.5_	Inhibitory Concentration 12.5
IC_25_	Inhibitory Concentration 25
IC_50_	Inhibitory Concentration 50
ICAM-1	Intercellular Adhesion Molecule-1
Km	Michaelis Constant
LFA-1	Lymphocyte Function-Associated Antigen-1
MALDI-TOF MS	Matrix-Assisted Laser Desorption/Ionization Time-of-Flight Mass Spectrometry
MD	Molecular Dynamic
mFBP	Membrane Folate-Binding Protein
MTT	Microculture Tetrazolium Technique/3-(4,5-dimethylthiazol-2-yl)-2,5-diphenyltetrazolium bromide
MTX	Methotrexate
MTXGlu	Polyglutamate Derivative of MTX
PBS	Phosphate-Buffered Saline
pH	Potential of Hydrogen
PI	Propidium Iodide
qRT-PCR	Quantitative-Reverse Transcriptase-Polymerase Chain Reaction
RFC	Reduced Folate Carrier
RNA	Ribonucleic Acid
RP-HPLC	Reversed Phase-High Performance Liquid Chromatography
RPMI	Roswell Park Memorial Institute
S Phase	Synthesis Phase
SD	Standard Deviation
SDS	Sodium Dodecyl Sulfate
SI	Selectivity Index
t1/2	Half Time
TR	Retention Time
*TYMS*	Thymidylate Synthase
VINALS	Vina Local Search
YASARA	Yet Another Scientific Artificial Reality Application
