# Supplementary material for: Selectively Targeting Leukemic MOLT-4 Cells by MTX-cIBR Conjugate: Mechanism of Action and Cellular Entry
Source: Life (Basel). 2026 Jun 11;16(6):981. doi: 10.3390/life16060981 (PMC13301118; doi:10.3390/life16060981)
Supplement: Supplementary file 1 [file life-16-00981-s001.zip › life-4310543-supplementary.pdf]

Supplementary Materials

Selectively Targeting Leukemic MOLT-4 cells by MTX-cIBR Conjugate: Mechanism of Action and Cellular Entry

Sista Werdyani,[1,2,3] Meagan E. Weldele,[3] Enade P. Istyastono,[4] Sofia M. Harjana,[5] Adi Hermawansyah,[6] Wariya Niraconkul,[7] Dewi K. Paramita,[5,8,*] and Teruna J. Siahaan[3,*]

(A)

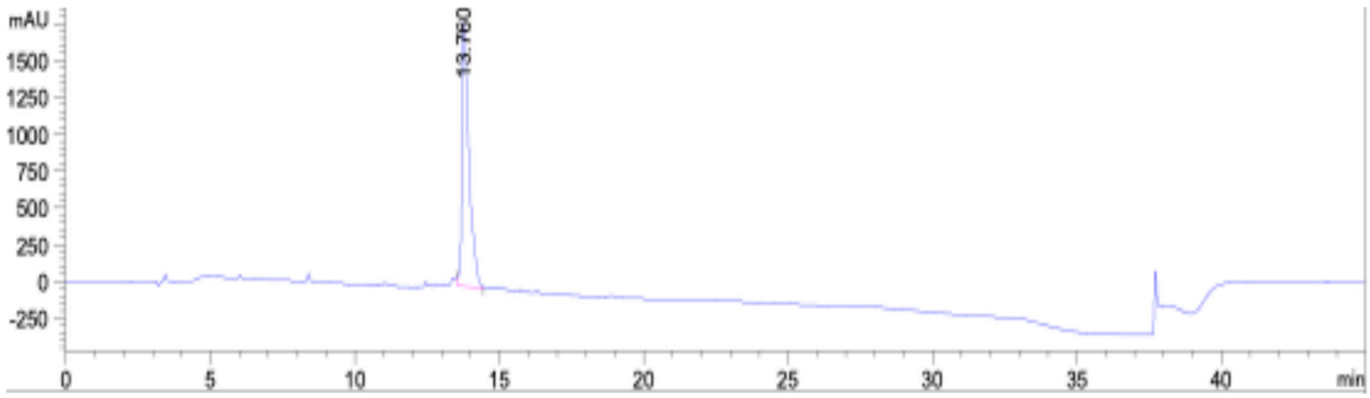

(B)

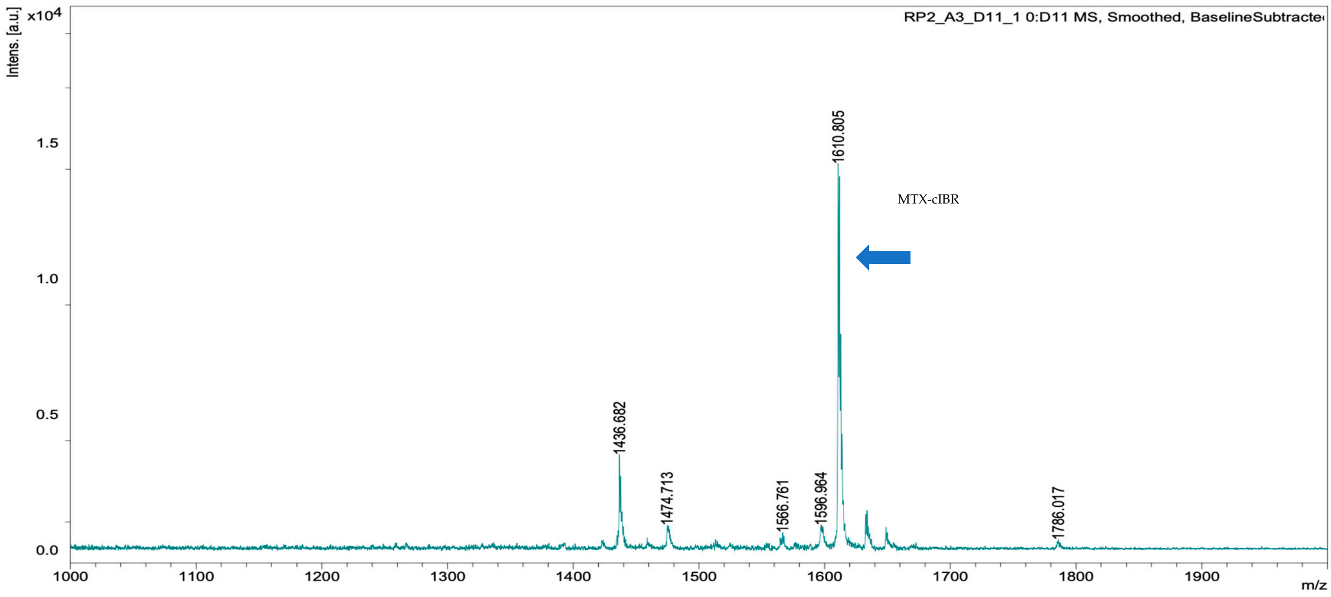

Supplementary Figure S1. (A) The RP-HPLC chromatogram of MTX-cIBR show a single peak to indicate its purity. **(B)** The spectrum of MALDI-TOF mass spectrometry for MTX-cIBR confirms the main at 1610 Da that corresponds to MTX-cIBR and the two larger peaks are from (M + Na^+) and (M + K^+) adducts.

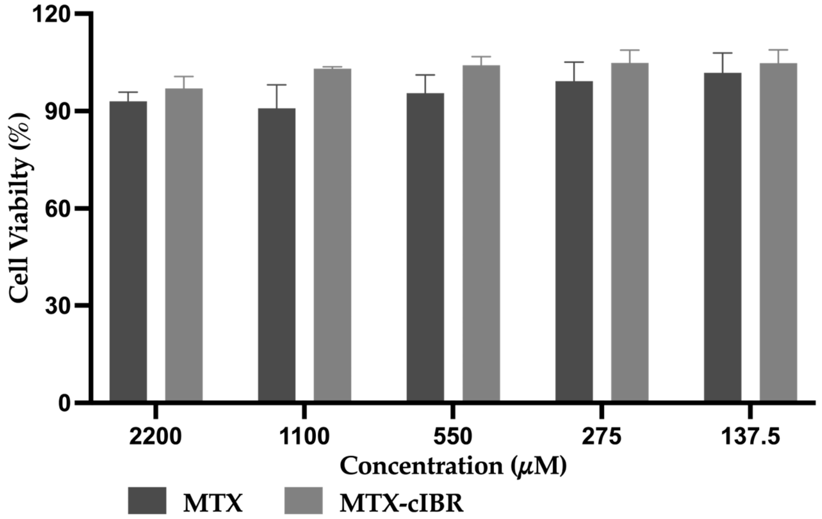

Supplementary Figure S2. Cell viability (%) of human dermal fibroblast cells following treatment with MTX and MTX-cIBR. Cell viability remained close to 100% at all tested concentrations, indicating minimal or no cytotoxic effects toward normal cells.

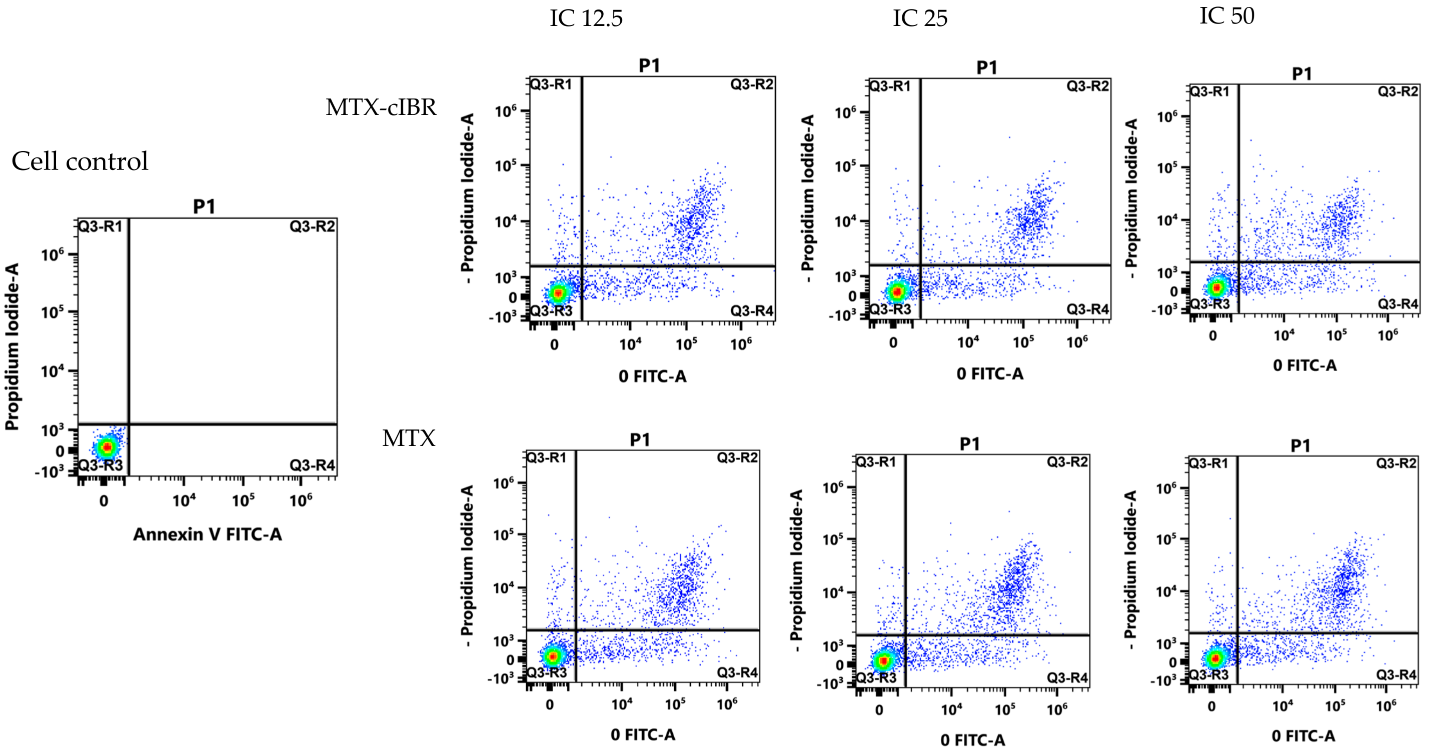

Supplementary Figure S3. Representative flow cytometry plots of apoptosis analysis in MOLT-4 cells treated with MTX and MTX-cIBR at $IC_{12.5}$, IC_{25}, and IC_{50} concentrations following Annexin V-FITC/PI staining.
